# Crystal structure of 2,4,6-tris­(cyclo­hex­yloxy)-1,3,5-triazine

**DOI:** 10.1107/S2056989015018782

**Published:** 2015-10-14

**Authors:** Ravish Sankolli, Jürg Hauser, T. N. Guru Row, Jürg Hulliger

**Affiliations:** a Department of Chemistry and Biochemistry, University of Bern, Freiestrasse 3, CH-3012 Bern, Switzerland; b Solid State and Structural Chemistry Unit, Indian Institute of Science, Bangalore 560 012, Karnataka, India

**Keywords:** crystal structure, triazine, cyclo­hexa­nol, channel inclusion, Piedfort units, hydrogen bonding

## Abstract

The title compound, C_21_H_33_N_3_O_3_, is a tri-substituted cyclo­hex­yloxy triazine. In the crystal, the triazine rings form (C3i-PU) Piedfort units. The inter-centroid distance of the π–π inter­action involving the triazine rings is 3.3914 (10) Å. In the crystal, mol­ecules are linked by C—H⋯O hydrogen bonds, forming ribbons propagating along [1-10]. There are also weak C—H⋯N and C—H⋯O contacts present, linking inversion-related ribbons, forming a three-dimensional structure.

## Abstract 2

The title compound, is the first tri-substituted cyclo­hex­yloxy triazine to be described. In the crystal, the triazine rings form (C3i-PU) Piedfort units and the mol­ecules are linked by C—H⋯O hydrogen bonds, forming ribbons propagating along [1-10].

## Chemical content   

Cyclo­hexyl derivatives are known to have applications in various fields of chemistry. The mono- and di-substituted derivatives of triazine with cyclo­hexa­nol show anti­viral activity (Mibu *et al.*, 2013 ▸), wherein cyclo­hexyl esters show the properties of traction fluids (Baldwin *et al.*, 1997 ▸). Partially substituted menth­oxy triazines can be used as enantio-differentiating reagents in organic synthesis (Kamiński *et al.*, 1998 ▸). The cyclo­hexyl trimer, perhydro­triphenelene (PHTP) can form inclusion compounds showing non-linear optical properties (Hoss *et al.*, 1996 ▸). In particular, PHTP as a renowned host in the literature, forms variable inclusions with functional mol­ecules (Allegra *et al.*, 1967 ▸; König *et al.*, 1997 ▸; Couderc & Hulliger, 2010 ▸). Most triazines also exhibit various types of inclusion properties (Süss *et al.*, 2002 ▸, 2005 ▸; Reichenbächer *et al.*, 2004 ▸). Thus, the title compound was synthesized to study the supra­molecular features in comparison to PHTP. Symmetrically substituted triazines with three cyclo­hexa­nol units through an oxygen linkage shows a trigonal symmetry in its *trans* racemic form and a planar geometry in its crystal structure. So far, the crystallization of the title compound with conventional solvents did not form any inclusions. To the best of our knowledge, this is the first tri-substituted cyclo­hex­yloxy triazine to be described.
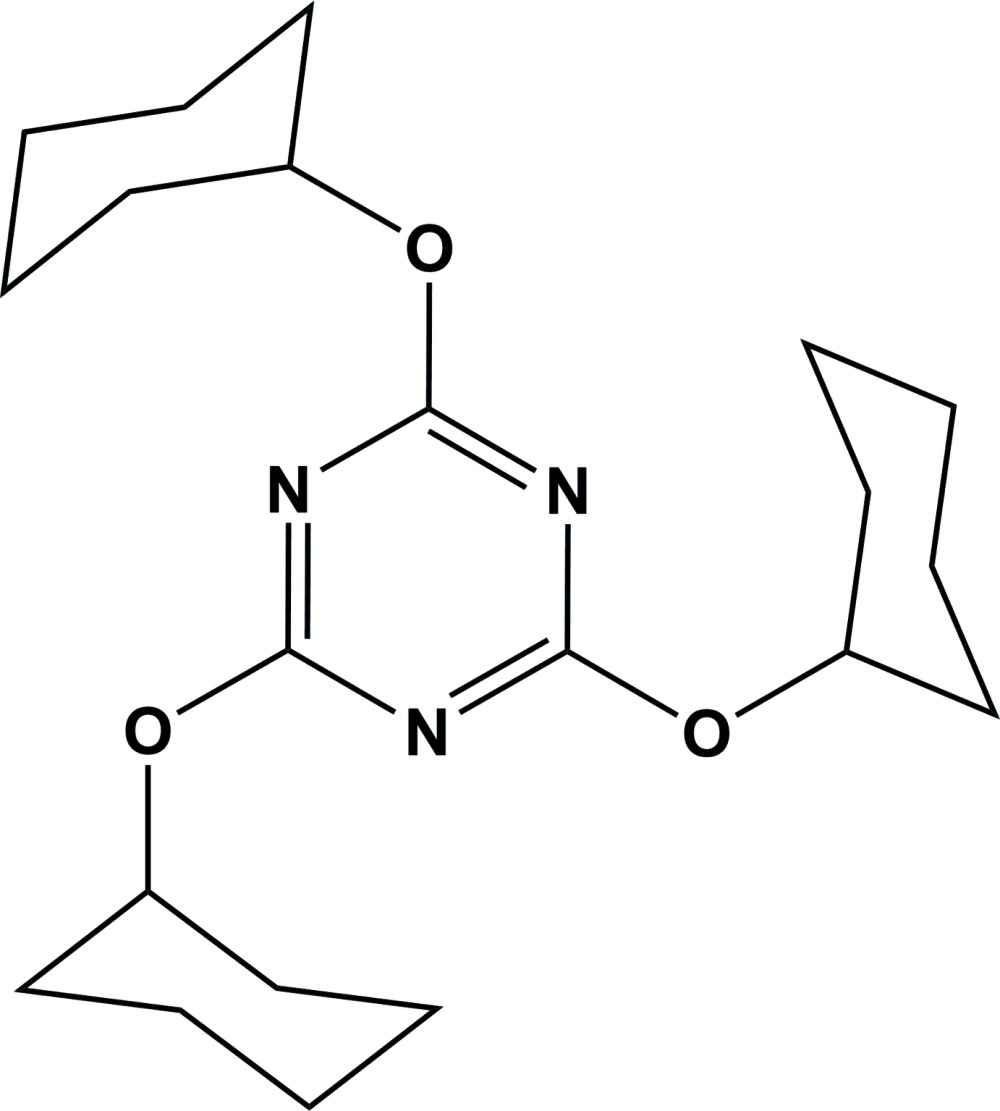


## Structural commentary   

**Figure 1:**
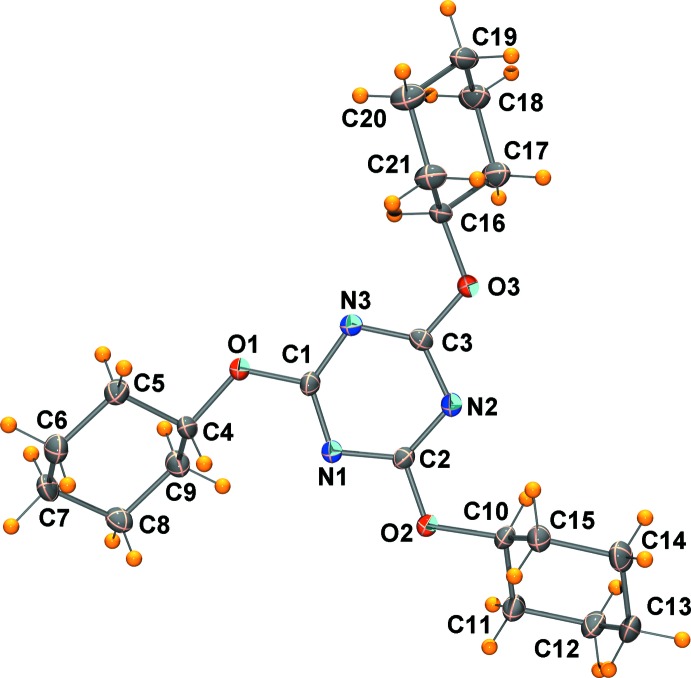
The mol­ecular structure of the title compound, with atom labelling. Displacement ellipsoids drawn at the 50% probability level. The C—O—C=N torsion angles are C4—O1—C1—N1 = 3.6 (2), C10—O2—C2—N2 = −1.2 (2) and C16—O3—C3—N3 = −3.1 (2)°.

The mol­ecular structure of the title compound is illustrated in Fig. 1 ▸. The mol­ecule has threefold rotation symmetry, but there are small variation in the C—O—C=N torsion angles; C4—O1—C1—N1 = 3.6 (2), C10—O2—C2—N2 = −1.2 (2) and C16—O3—C3—N3 = −3.1 (2)°.

## Supra­molecular features   

**Figure 2:**
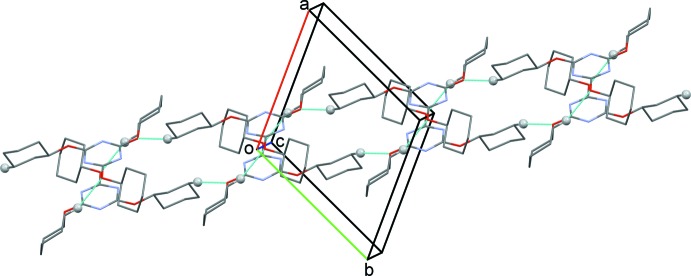
A view along the *c* axis of the crystal packing of the title compound. The most significant C—H⋯O hydrogen bonds (see Table 1 ▸) are shown as dashed lines, and the only H atoms shown are H12*A* and H9*A* (grey balls) for clarity.

**Figure 3:**
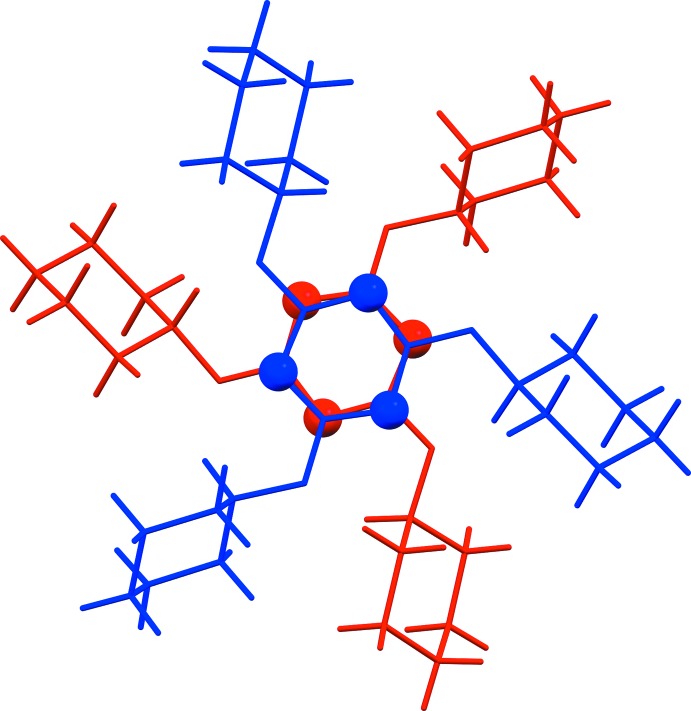
A view of the Piedfort unit (C3i-PU), with the two triazine rings stacking one above the other, forming an hexa­gonal symmetry unit. The N atoms are shown as red and blue balls.

**Figure 4:**
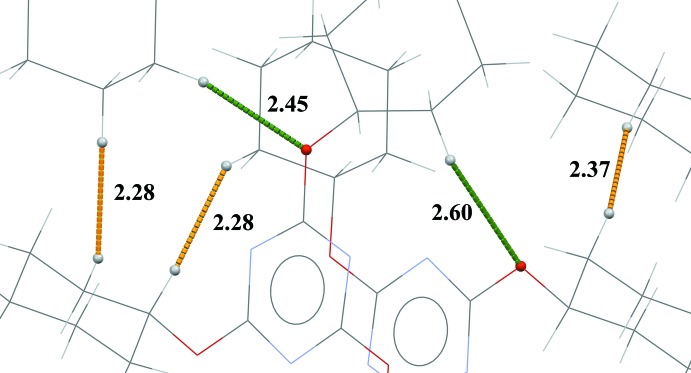
A view of the short C—H⋯H⋯C contacts (orange dashed lines) and some C—H⋯O hydrogen bonds (green dashed lines; see Table 1 ▸) in the crystal structure of the title compound.

In the crystal, mol­ecules are linked by C—H⋯O hydrogen bonds, forming ribbons propagating along [1
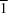
0] (Fig. 2 ▸ and Table 1 ▸). Inversion-related ribbons are linked by weak C—H⋯N and C—H⋯O contacts, forming a three-dimensional structure (Table 1 ▸). There are Piedfort units (C3i-PU) present (Jessiman *et al.*, 1990 ▸), as shown in Fig. 3 ▸. The inter-centroid distance of the slightly slipped parallel π–π inter­action involving inversion-related triazine rings is 3.3914 (10) Å. The inter-planar distance is 3.3315 (7) Å, while the slippage is 0.634 Å. There are three C—H⋯H—C van der Waals contacts, 2.28, 2.28 and 2.37 Å (Fig. 4 ▸), which are longer than those in the crystal structure of PHTP (measured 2.13, 2.14 and 2.16 Å; Harlow & Desiraju, 1990 ▸).

The perhydrogenated outer wall resembles the structural features of PHTP (pe­hydro­tri­phenyl­ene) in its crystal structure with C—H⋯H—C short contacts (Harlow & Desiraju, 1990 ▸). In comparison, PHTP is a highly symmetrical chiral mol­ecule, which is used for inclusions in its all-*trans* racemic form (König *et al.*, 1997 ▸). Thus, the title compound is a perhydrogenated triazine analogue of PHTP. However, the triazine rings which form Piedfort units (Jessiman *et al.*, 1990 ▸) and the C—H⋯O and C—H⋯N hydrogen bonds (Table 1 ▸) contribute to the stabilization of the structure as compared to PHTP.

## Synthesis and crystallization   

Cyclo­hexa­nol (10.4 ml, 10.02 g, 100 mmol) and sodium hydride (2.88 g, 120 mmol) were taken in a round bottom flask containing 50 ml of THF at 273 K. The mixture was stirred at room temperature for 30 min, then cyanuric chloride (4.6 g, 25 mmol) was carefully added in one portion. The mixture was stirred overnight at 323 K. The solvent was then removed under reduced pressure and the oily mixture was transferred in to a separating funnel and extracted with CH_2_Cl_2_ (3 × 100 ml). Again, the solvent was removed under reduced pressure and the crude product was further purified through column chromatography (SiO_2_ 60, eluent: diethyl ether/pentane 1:1) to yield the pure product as a white powder. Colourless prismatic crystals were obtained by isothermal evaporation of a solution in THF.

## Refinement   

Crystal data, data collection and structure refinement details are summarized in Table 2 ▸. The C-bound H atoms were included in calculated positions and treated as riding atoms: C—H = 0.99–1.00 Å with *U*_iso_(H) = 1.2*U*_eq_(C).

## Supplementary Material

Crystal structure: contains datablock(s) I. DOI: 10.1107/S2056989015018782/su5213sup1.cif

Structure factors: contains datablock(s) I. DOI: 10.1107/S2056989015018782/su5213Isup2.hkl

Supporting information file. DOI: 10.1107/S2056989015018782/su5213Isup3.cml

CCDC reference: 1430153

Additional supporting information:  crystallographic information; 3D view; checkCIF report

## Figures and Tables

**Table 1:** Hydrogen-bond geometry (, )

*D*H*A*	*D*H	H*A*	*D* *A*	*D*H*A*
C12H12*A*O1^i^	0.99	2.45	3.413(2)	164
C9H9*A*O3^ii^	0.99	2.60	3.528(2)	156
C10H10O1^ii^	1.00	2.95	3.787(2)	142
C5H5*B*N1^iii^	0.99	2.77	3.684(2)	154

Symmetry codes: (i) 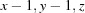; (ii) 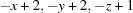; (iii) 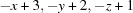.

**Table 2:** Experimental details

Crystal data	
Chemical formula	C_21_H_33_N_3_O_3_
*M*_r_	375.50
Crystal system, space group	Triclinic, *P* 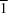
Temperature (K)	100
*a*, *b*, *c* ()	9.7020(2), 10.1456(3), 11.2064(3)
, , ()	96.528(2), 95.982(2), 112.110(2)
*V* (^3^)	1002.30(5)
*Z*	2
Radiation type	Mo *K*
(mm^1^)	0.08
Crystal size (mm)	0.47 0.24 0.10

Data collection	
Diffractometer	Agilent SuperNova, Eos
Absorption correction	Multi-scan (*CrysAlis PRO*; Agilent, 2014 ▸)
*T*_min_, *T*_max_	0.657, 1
No. of measured, independent and observed [*I* > 2(*I*)] reflections	24791, 4106, 3603
*R*_int_	0.027
(sin /)_max_ (^1^)	0.625

Refinement	
*R*[*F* ^2^ > 2(*F* ^2^)], *wR*(*F* ^2^), *S*	0.052, 0.142, 1.04
No. of reflections	4106
No. of parameters	244
H-atom treatment	H-atom parameters constrained
_max_, _min_ (e ^3^)	0.61, 0.21

Computer programs: *CrysAlis PRO* (Agilent, 2014 ▸), *SHELXS2014*/7 (Sheldrick, 2008 ▸), *SHELXL2014*/7 (Sheldrick, 2015 ▸), *POV-RAY* (*POV-RAY* Team, 2004 ▸), *Mercury* (Macrae *et al.*, 2008 ▸), and *PLATON* (Spek, 2009 ▸).

## supplementary crystallographic information

### Crystal data

**Table:**

C_21_H_33_N_3_O_3_	*Z* = 2
*M**_r_* = 375.50	*F*(000) = 408
Triclinic, *P*1	*D*_x_ = 1.244 Mg m^−^^3^
*a* = 9.7020 (2) Å	Mo *K*α radiation, λ = 0.71073 Å
*b* = 10.1456 (3) Å	Cell parameters from 9471 reflections
*c* = 11.2064 (3) Å	θ = 2.2–27.7°
α = 96.528 (2)°	µ = 0.08 mm^−^^1^
β = 95.982 (2)°	*T* = 100 K
γ = 112.110 (2)°	Prism, colourless
*V* = 1002.30 (5) Å^3^	0.47 × 0.24 × 0.10 mm

### Data collection

**Table:**

Agilent SuperNova, Eos diffractometer	4106 independent reflections
Radiation source: Mo X-ray Source	3603 reflections with *I* > 2σ(*I*)
Detector resolution: 16.0965 pixels mm^-1^	*R*_int_ = 0.027
ω scans	θ_max_ = 26.4°, θ_min_ = 1.9°
Absorption correction: multi-scan (*CrysAlis PRO*; Agilent, 2014)	*h* = −12→12
*T*_min_ = 0.657, *T*_max_ = 1	*k* = −12→12
24791 measured reflections	*l* = −13→13

### Refinement

**Table:**

Refinement on *F*^2^	0 restraints
Least-squares matrix: full	Hydrogen site location: inferred from neighbouring sites
*R*[*F*^2^ > 2σ(*F*^2^)] = 0.052	H-atom parameters constrained
*wR*(*F*^2^) = 0.142	*w* = 1/[σ^2^(*F*_o_^2^) + (0.0651*P*)^2^ + 0.7913*P*] where *P* = (*F*_o_^2^ + 2*F*_c_^2^)/3
*S* = 1.04	(Δ/σ)_max_ < 0.001
4106 reflections	Δρ_max_ = 0.61 e Å^−^^3^
244 parameters	Δρ_min_ = −0.21 e Å^−^^3^

### Special details

**Table:**

Geometry. All e.s.d.'s (except the e.s.d. in the dihedral angle between two l.s. planes) are estimated using the full covariance matrix. The cell e.s.d.'s are taken into account individually in the estimation of e.s.d.'s in distances, angles and torsion angles; correlations between e.s.d.'s in cell parameters are only used when they are defined by crystal symmetry. An approximate (isotropic) treatment of cell e.s.d.'s is used for estimating e.s.d.'s involving l.s. planes.

### Fractional atomic coordinates and isotropic or equivalent isotropic displacement parameters (Å^2^)

**Table:**

	*x*	*y*	*z*	*U*_iso_*/*U*_eq_	
C1	1.24843 (18)	1.05014 (17)	0.49190 (14)	0.0195 (3)	
C2	1.05411 (17)	0.83773 (16)	0.45059 (14)	0.0188 (3)	
C3	1.07636 (18)	1.00085 (17)	0.32811 (14)	0.0194 (3)	
C4	1.44642 (19)	1.10652 (17)	0.66284 (14)	0.0225 (4)	
H4	1.4372	1.0047	0.6426	0.027*	
C5	1.61162 (19)	1.20826 (19)	0.68357 (16)	0.0256 (4)	
H5A	1.6204	1.3094	0.6976	0.031*	
H5B	1.6569	1.1941	0.6105	0.031*	
C6	1.6957 (2)	1.1792 (2)	0.79337 (17)	0.0295 (4)	
H6A	1.6956	1.0813	0.7755	0.035*	
H6B	1.8018	1.2495	0.8091	0.035*	
C7	1.6240 (2)	1.19059 (19)	0.90564 (16)	0.0284 (4)	
H7A	1.6353	1.2916	0.9295	0.034*	
H7B	1.6765	1.1642	0.9737	0.034*	
C8	1.4568 (2)	1.0913 (2)	0.88254 (16)	0.0277 (4)	
H8A	1.4459	0.9896	0.8677	0.033*	
H8B	1.4114	1.1055	0.9556	0.033*	
C9	1.3732 (2)	1.1221 (2)	0.77312 (16)	0.0271 (4)	
H9A	1.2663	1.0536	0.7574	0.033*	
H9B	1.3763	1.2212	0.7902	0.033*	
C10	0.83931 (18)	0.61278 (17)	0.40889 (15)	0.0220 (3)	
H10	0.7850	0.6696	0.3740	0.026*	
C11	0.7472 (2)	0.5209 (2)	0.49280 (16)	0.0293 (4)	
H11A	0.7268	0.5829	0.5575	0.035*	
H11B	0.8038	0.4697	0.5319	0.035*	
C12	0.5979 (2)	0.4112 (2)	0.41799 (17)	0.0330 (4)	
H12A	0.5375	0.3498	0.4718	0.040*	
H12B	0.5395	0.4630	0.3826	0.040*	
C13	0.6271 (2)	0.31714 (19)	0.31667 (17)	0.0317 (4)	
H13A	0.5300	0.2477	0.2689	0.038*	
H13B	0.6814	0.2618	0.3520	0.038*	
C14	0.7213 (2)	0.4114 (2)	0.23329 (16)	0.0297 (4)	
H14A	0.6643	0.4622	0.1941	0.036*	
H14B	0.7422	0.3498	0.1685	0.036*	
C15	0.87075 (19)	0.52219 (18)	0.30727 (15)	0.0243 (4)	
H15A	0.9311	0.4715	0.3417	0.029*	
H15B	0.9295	0.5851	0.2535	0.029*	
C16	1.09447 (19)	1.17766 (17)	0.19788 (15)	0.0219 (3)	
H16	1.1439	1.2490	0.2743	0.026*	
C17	0.9714 (2)	1.2129 (2)	0.13237 (19)	0.0314 (4)	
H17A	0.8992	1.2163	0.1877	0.038*	
H17B	0.9159	1.1367	0.0612	0.038*	
C18	1.0407 (2)	1.3590 (2)	0.09008 (19)	0.0338 (4)	
H18A	0.9602	1.3788	0.0434	0.041*	
H18B	1.0872	1.4361	0.1620	0.041*	
C19	1.1586 (2)	1.36119 (19)	0.01149 (16)	0.0310 (4)	
H19A	1.2046	1.4582	−0.0110	0.037*	
H19B	1.1103	1.2906	−0.0643	0.037*	
C20	1.2807 (2)	1.3241 (2)	0.07840 (19)	0.0350 (4)	
H20A	1.3352	1.3995	0.1503	0.042*	
H20B	1.3542	1.3216	0.0241	0.042*	
C21	1.2106 (2)	1.17700 (19)	0.11921 (17)	0.0292 (4)	
H21A	1.1629	1.1006	0.0469	0.035*	
H21B	1.2903	1.1557	0.1653	0.035*	
N1	1.18158 (15)	0.92270 (14)	0.52567 (12)	0.0202 (3)	
N2	0.99553 (15)	0.86893 (14)	0.35006 (12)	0.0203 (3)	
N3	1.20286 (15)	1.09706 (14)	0.39485 (12)	0.0208 (3)	
O1	1.37668 (13)	1.14586 (12)	0.55837 (10)	0.0244 (3)	
O2	0.98237 (13)	0.71122 (12)	0.48406 (10)	0.0227 (3)	
O3	1.01921 (13)	1.03331 (12)	0.22770 (10)	0.0230 (3)	

### Atomic displacement parameters (Å^2^)

**Table:**

	*U*^11^	*U*^22^	*U*^33^	*U*^12^	*U*^13^	*U*^23^
C1	0.0179 (7)	0.0191 (8)	0.0164 (7)	0.0030 (6)	0.0012 (6)	−0.0001 (6)
C2	0.0186 (7)	0.0166 (7)	0.0189 (7)	0.0042 (6)	0.0042 (6)	0.0024 (6)
C3	0.0224 (8)	0.0219 (8)	0.0154 (7)	0.0098 (6)	0.0041 (6)	0.0042 (6)
C4	0.0239 (8)	0.0199 (8)	0.0180 (8)	0.0039 (7)	−0.0038 (6)	0.0036 (6)
C5	0.0223 (8)	0.0265 (9)	0.0236 (8)	0.0046 (7)	0.0038 (7)	0.0047 (7)
C6	0.0204 (8)	0.0332 (10)	0.0304 (9)	0.0062 (7)	−0.0004 (7)	0.0063 (7)
C7	0.0308 (9)	0.0273 (9)	0.0211 (8)	0.0080 (7)	−0.0067 (7)	0.0020 (7)
C8	0.0290 (9)	0.0344 (10)	0.0206 (8)	0.0120 (8)	0.0051 (7)	0.0091 (7)
C9	0.0220 (8)	0.0340 (9)	0.0238 (9)	0.0089 (7)	0.0023 (7)	0.0075 (7)
C10	0.0201 (8)	0.0168 (8)	0.0218 (8)	0.0004 (6)	0.0006 (6)	0.0019 (6)
C11	0.0282 (9)	0.0278 (9)	0.0198 (8)	−0.0018 (7)	0.0013 (7)	0.0032 (7)
C12	0.0284 (9)	0.0279 (9)	0.0286 (9)	−0.0044 (7)	0.0026 (7)	0.0056 (7)
C13	0.0323 (10)	0.0191 (8)	0.0309 (9)	−0.0001 (7)	−0.0077 (8)	0.0008 (7)
C14	0.0334 (10)	0.0279 (9)	0.0226 (8)	0.0104 (8)	−0.0032 (7)	−0.0032 (7)
C15	0.0238 (8)	0.0244 (8)	0.0208 (8)	0.0066 (7)	0.0006 (6)	0.0014 (6)
C16	0.0260 (8)	0.0182 (8)	0.0186 (8)	0.0055 (6)	0.0007 (6)	0.0054 (6)
C17	0.0253 (9)	0.0287 (9)	0.0407 (11)	0.0093 (7)	0.0038 (8)	0.0138 (8)
C18	0.0337 (10)	0.0292 (10)	0.0416 (11)	0.0135 (8)	0.0046 (8)	0.0152 (8)
C19	0.0426 (11)	0.0223 (8)	0.0223 (8)	0.0061 (8)	0.0024 (8)	0.0076 (7)
C20	0.0330 (10)	0.0318 (10)	0.0415 (11)	0.0098 (8)	0.0145 (8)	0.0143 (8)
C21	0.0306 (9)	0.0261 (9)	0.0344 (10)	0.0122 (7)	0.0099 (8)	0.0110 (7)
N1	0.0195 (7)	0.0196 (7)	0.0167 (6)	0.0031 (5)	−0.0001 (5)	0.0030 (5)
N2	0.0191 (7)	0.0202 (7)	0.0177 (6)	0.0046 (5)	0.0003 (5)	0.0018 (5)
N3	0.0228 (7)	0.0183 (7)	0.0175 (7)	0.0041 (6)	0.0019 (5)	0.0038 (5)
O1	0.0228 (6)	0.0209 (6)	0.0204 (6)	−0.0001 (5)	−0.0041 (5)	0.0056 (5)
O2	0.0220 (6)	0.0188 (6)	0.0202 (6)	0.0009 (5)	−0.0012 (4)	0.0041 (4)
O3	0.0233 (6)	0.0203 (6)	0.0202 (6)	0.0037 (5)	−0.0025 (5)	0.0051 (4)

### Geometric parameters (Å, º)

**Table:**

C1—N1	1.329 (2)	C11—H11A	0.9900
C1—O1	1.3338 (19)	C11—H11B	0.9900
C1—N3	1.334 (2)	C12—C13	1.518 (3)
C2—O2	1.3281 (19)	C12—H12A	0.9900
C2—N2	1.333 (2)	C12—H12B	0.9900
C2—N1	1.340 (2)	C13—C14	1.532 (3)
C3—N3	1.326 (2)	C13—H13A	0.9900
C3—O3	1.3318 (19)	C13—H13B	0.9900
C3—N2	1.339 (2)	C14—C15	1.537 (2)
C4—O1	1.4601 (19)	C14—H14A	0.9900
C4—C9	1.510 (2)	C14—H14B	0.9900
C4—C5	1.520 (2)	C15—H15A	0.9900
C4—H4	1.0000	C15—H15B	0.9900
C5—C6	1.524 (2)	C16—O3	1.4652 (19)
C5—H5A	0.9900	C16—C21	1.502 (2)
C5—H5B	0.9900	C16—C17	1.514 (2)
C6—C7	1.513 (3)	C16—H16	1.0000
C6—H6A	0.9900	C17—C18	1.533 (2)
C6—H6B	0.9900	C17—H17A	0.9900
C7—C8	1.529 (2)	C17—H17B	0.9900
C7—H7A	0.9900	C18—C19	1.510 (3)
C7—H7B	0.9900	C18—H18A	0.9900
C8—C9	1.526 (2)	C18—H18B	0.9900
C8—H8A	0.9900	C19—C20	1.524 (3)
C8—H8B	0.9900	C19—H19A	0.9900
C9—H9A	0.9900	C19—H19B	0.9900
C9—H9B	0.9900	C20—C21	1.535 (2)
C10—O2	1.4680 (18)	C20—H20A	0.9900
C10—C15	1.510 (2)	C20—H20B	0.9900
C10—C11	1.516 (2)	C21—H21A	0.9900
C10—H10	1.0000	C21—H21B	0.9900
C11—C12	1.536 (2)		
			
N1—C1—O1	119.36 (14)	H12A—C12—H12B	108.1
N1—C1—N3	127.33 (14)	C12—C13—C14	109.90 (15)
O1—C1—N3	113.31 (14)	C12—C13—H13A	109.7
O2—C2—N2	119.16 (14)	C14—C13—H13A	109.7
O2—C2—N1	114.21 (14)	C12—C13—H13B	109.7
N2—C2—N1	126.63 (14)	C14—C13—H13B	109.7
N3—C3—O3	119.32 (14)	H13A—C13—H13B	108.2
N3—C3—N2	126.75 (14)	C13—C14—C15	110.08 (14)
O3—C3—N2	113.93 (14)	C13—C14—H14A	109.6
O1—C4—C9	111.01 (14)	C15—C14—H14A	109.6
O1—C4—C5	105.24 (13)	C13—C14—H14B	109.6
C9—C4—C5	111.64 (14)	C15—C14—H14B	109.6
O1—C4—H4	109.6	H14A—C14—H14B	108.2
C9—C4—H4	109.6	C10—C15—C14	109.76 (14)
C5—C4—H4	109.6	C10—C15—H15A	109.7
C4—C5—C6	109.90 (14)	C14—C15—H15A	109.7
C4—C5—H5A	109.7	C10—C15—H15B	109.7
C6—C5—H5A	109.7	C14—C15—H15B	109.7
C4—C5—H5B	109.7	H15A—C15—H15B	108.2
C6—C5—H5B	109.7	O3—C16—C21	109.68 (13)
H5A—C5—H5B	108.2	O3—C16—C17	105.89 (13)
C7—C6—C5	111.39 (15)	C21—C16—C17	111.75 (14)
C7—C6—H6A	109.3	O3—C16—H16	109.8
C5—C6—H6A	109.3	C21—C16—H16	109.8
C7—C6—H6B	109.3	C17—C16—H16	109.8
C5—C6—H6B	109.3	C16—C17—C18	109.82 (15)
H6A—C6—H6B	108.0	C16—C17—H17A	109.7
C6—C7—C8	111.16 (14)	C18—C17—H17A	109.7
C6—C7—H7A	109.4	C16—C17—H17B	109.7
C8—C7—H7A	109.4	C18—C17—H17B	109.7
C6—C7—H7B	109.4	H17A—C17—H17B	108.2
C8—C7—H7B	109.4	C19—C18—C17	111.37 (16)
H7A—C7—H7B	108.0	C19—C18—H18A	109.4
C9—C8—C7	111.15 (14)	C17—C18—H18A	109.4
C9—C8—H8A	109.4	C19—C18—H18B	109.4
C7—C8—H8A	109.4	C17—C18—H18B	109.4
C9—C8—H8B	109.4	H18A—C18—H18B	108.0
C7—C8—H8B	109.4	C18—C19—C20	110.83 (15)
H8A—C8—H8B	108.0	C18—C19—H19A	109.5
C4—C9—C8	109.41 (14)	C20—C19—H19A	109.5
C4—C9—H9A	109.8	C18—C19—H19B	109.5
C8—C9—H9A	109.8	C20—C19—H19B	109.5
C4—C9—H9B	109.8	H19A—C19—H19B	108.1
C8—C9—H9B	109.8	C19—C20—C21	110.28 (16)
H9A—C9—H9B	108.2	C19—C20—H20A	109.6
O2—C10—C15	109.31 (13)	C21—C20—H20A	109.6
O2—C10—C11	106.48 (13)	C19—C20—H20B	109.6
C15—C10—C11	111.73 (14)	C21—C20—H20B	109.6
O2—C10—H10	109.8	H20A—C20—H20B	108.1
C15—C10—H10	109.8	C16—C21—C20	110.14 (15)
C11—C10—H10	109.8	C16—C21—H21A	109.6
C10—C11—C12	108.91 (14)	C20—C21—H21A	109.6
C10—C11—H11A	109.9	C16—C21—H21B	109.6
C12—C11—H11A	109.9	C20—C21—H21B	109.6
C10—C11—H11B	109.9	H21A—C21—H21B	108.1
C12—C11—H11B	109.9	C1—N1—C2	112.84 (13)
H11A—C11—H11B	108.3	C2—N2—C3	113.31 (14)
C13—C12—C11	110.63 (16)	C3—N3—C1	113.12 (13)
C13—C12—H12A	109.5	C1—O1—C4	119.74 (12)
C11—C12—H12A	109.5	C2—O2—C10	117.91 (12)
C13—C12—H12B	109.5	C3—O3—C16	118.62 (12)
C11—C12—H12B	109.5		
			
C4—O1—C1—N1	3.6 (2)	O1—C4—C5—C6	178.67 (14)
C4—O1—C1—N3	−175.80 (14)	C9—C4—C5—C6	58.2 (2)
C1—O1—C4—C5	157.44 (15)	O1—C4—C9—C8	−175.68 (14)
C1—O1—C4—C9	−81.64 (18)	C5—C4—C9—C8	−58.60 (19)
C2—O2—C10—C15	87.08 (17)	C4—C5—C6—C7	−55.8 (2)
C10—O2—C2—N1	178.32 (14)	C5—C6—C7—C8	55.0 (2)
C10—O2—C2—N2	−1.2 (2)	C6—C7—C8—C9	−55.5 (2)
C2—O2—C10—C11	−152.09 (15)	C7—C8—C9—C4	56.8 (2)
C3—O3—C16—C17	−148.75 (15)	O2—C10—C11—C12	−177.83 (14)
C16—O3—C3—N2	177.09 (15)	C11—C10—C15—C14	58.48 (19)
C16—O3—C3—N3	−3.1 (2)	C15—C10—C11—C12	−58.6 (2)
C3—O3—C16—C21	90.51 (18)	O2—C10—C15—C14	176.06 (13)
C2—N1—C1—O1	−179.98 (15)	C10—C11—C12—C13	58.4 (2)
C1—N1—C2—O2	−178.09 (15)	C11—C12—C13—C14	−58.9 (2)
C2—N1—C1—N3	−0.7 (3)	C12—C13—C14—C15	58.0 (2)
C1—N1—C2—N2	1.4 (3)	C13—C14—C15—C10	−57.36 (19)
C2—N2—C3—N3	0.1 (3)	O3—C16—C17—C18	−176.46 (14)
C3—N2—C2—O2	178.35 (15)	C21—C16—C17—C18	−57.1 (2)
C3—N2—C2—N1	−1.1 (3)	O3—C16—C21—C20	175.18 (14)
C2—N2—C3—O3	179.86 (14)	C17—C16—C21—C20	58.1 (2)
C1—N3—C3—O3	−179.28 (15)	C16—C17—C18—C19	55.9 (2)
C3—N3—C1—N1	−0.2 (3)	C17—C18—C19—C20	−56.4 (2)
C3—N3—C1—O1	179.16 (15)	C18—C19—C20—C21	56.6 (2)
C1—N3—C3—N2	0.5 (3)	C19—C20—C21—C16	−57.1 (2)

### Hydrogen-bond geometry (Å, º)

**Table:**

*D*—H···*A*	*D*—H	H···*A*	*D*···*A*	*D*—H···*A*
C12—H12*A*···O1^i^	0.99	2.45	3.413 (2)	164
C9—H9*A*···O3^ii^	0.99	2.60	3.528 (2)	156
C10—H10···O1^ii^	1.00	2.95	3.787 (2)	142
C5—H5*B*···N1^iii^	0.99	2.77	3.684 (2)	154

Symmetry codes: (i) *x*−1, *y*−1, *z*; (ii) −*x*+2, −*y*+2, −*z*+1; (iii) −*x*+3, −*y*+2, −*z*+1.
